# Sol–Gel-Synthesized Pt, Ni and Co-Based Electrocatalyst Effects of the Support Type, Characterization, and Possible Application in AEM-URFC

**DOI:** 10.3390/gels11040229

**Published:** 2025-03-22

**Authors:** Elitsa Stanislavova Petkucheva, Borislava Mladenova, Mohsin Muhyuddin, Mariela Dimitrova, Galin Rusev Borisov, Carlo Santoro, Evelina Slavcheva

**Affiliations:** 1 “Acad. Evgeni Budevski” Institute of Electrochemistry and Energy Systems (IEES), Bulgarian Academy of Sciences (BAS), Acad. G. Bonchev Str., bl. 10, 1113 Sofia, Bulgaria; borislava.mladenova@iees.bas.bg (B.M.); mariela.dimitrova@iees.bas.bg (M.D.); gal.rusev@iees.bas.bg (G.R.B.); eslavcheva@iees.bas.bg (E.S.); 2 Electrocatalysis and Bioelectrocatalysis Laboratory (EBLab), Department of Materials Science, University of Milano-Bicocca, Building U5, Via Cozzi 55, 20125 Milan, Italy; m.muhyuddin@campus.unimib.it (M.M.); carlo.santoro@unimib.it (C.S.)

**Keywords:** sol–gel method, electocatalysts, bifunctional, Pt, Co, Ni, Magnelli phases titania, carbon, PEM-URFC

## Abstract

This study explores the synthesis and characterization of platinum (Pt), nickel (Ni), and cobalt (Co)-based electrocatalysts using the sol–gel method. The focus is on the effect of different support materials on the catalytic performance in alkaline media. The sol–gel technique enables the production of highly uniform electrocatalysts, supported on carbon-based substrates, metal oxides, and conductive polymers. Various characterization techniques, including X-ray diffraction (XRD) and scanning electron microscopy (SEM), were used to analyze the structure of the synthesized materials, while their electrochemical properties, which are relevant to their application in unitized regenerative fuel cells (URFCs), were investigated using cyclic voltammetry (CV) and linear sweep voltammetry (LSV). This hydrogen energy-converting device integrates water electrolyzers and fuel cells into a single system, reducing weight, volume, and cost. However, their performance is constrained by the electrocatalyst’s oxygen bifunctional activity. To improve URFC efficiency, an ideal electrocatalyst should exhibit high oxygen evolution (OER) and oxygen reduction (ORR) activity with a low bifunctionality index (BI). The present study evaluated the prepared electrocatalysts in an alkaline medium, finding that Pt25-Co75/XC72R and Pt75-Co25/N82 demonstrated promising bifunctional activity. The results suggest that these electrocatalysts are well-suited for both electrolysis and fuel cell operation in anion exchange membrane-unitized regenerative fuel cells (AEM-URFCs), contributing to improved round-trip efficiency.

## 1. Introduction

Hydrogen is a promising clean energy carrier that can efficiently utilize renewable energy sources while reducing greenhouse gas emissions. One key technology for hydrogen-based energy systems is unitized regenerative fuel cells (URFCs), which function both as water electrolyzers (to produce hydrogen) and fuel cells (to generate electricity). However, the commercialization of anion exchange membrane-URFCs (AEM-URFCs) faces challenges related to the performance, stability, and cost of electrocatalysts used for hydrogen and oxygen reactions [1,2,3,4,5,6,7,8,9,10].

The development of efficient and durable electrocatalysts is essential for advancing clean energy technologies such as fuel cells and water electrolysis. Precious metal-based electrocatalysts, particularly platinum (Pt), are widely recognized for their exceptional catalytic activity in hydrogen evolution reactions (HERs), hydrogen oxidation reactions (HORs), oxygen evolution reactions (OERs), and oxygen reduction reactions (ORRs) [11,12]. However, the high cost and limited availability of Pt drive research toward hybrid electrocatalysts that combine Pt with transition metals like cobalt (Co) and nickel (Ni) to enhance performance and stability while reducing noble metal content [13,14].

The choice of support material plays a significant role in determining the activity, stability, and overall performance of electrocatalysts. To further optimize electrocatalyst performance, researchers investigate different support materials, which influence electrocatalyst stability and efficiency. Various supports, such as carbon-based materials, metal oxides, and conductive polymers, are used to enhance the electrocatalytic behavior of these metals by improving electron transfer, preventing electrocatalyst sintering, and enhancing the overall conductivity of the system [15].

Carbon black (Vulcan XC72R) is commonly used as a support material for electrocatalysts due to its excellent electrical conductivity, high surface area, and ability to prevent metal particle aggregation, which are crucial for enhancing the performance and stability of the catalyst in electrochemical reactions [16,17,18]. The conductive network provided by carbon black facilitates efficient charge transfer, which is vital for the hydrogen evolution reaction (HER), oxygen reduction reaction (ORR), and hydrogen oxidation reaction (HOR). Moreover, its high surface area allows for a better dispersion of metal nanoparticles, reducing the risk of catalyst sintering and improving the long-term stability of the catalyst. These structural properties of carbon black contribute to improving reaction kinetics, especially under the harsh operating conditions of fuel cells and electrolyzers [19].

On the other hand, Magnéli-phase titania (MPT), with its unique structural and electronic properties, offers several advantages in catalytic reactions, especially under alkaline conditions. MPTs, such as Ti_4_O_7_ and Ti_5_O_9_, exhibit metallic-like conductivity, which allows for efficient electron transport, and their high thermal stability and redox-active nature make them resistant to oxidation and degradation under harsh conditions typically encountered in oxygen evolution reactions (OERs) and ORRs [20,21,22,23,24,25,26,27]. Furthermore, the corrosion resistance and tunable electronic properties of MPT enhance the interaction between the metal nanoparticles (Pt, Ni, and Co) and the support material, improving both the catalytic efficiency and the long-term stability of the catalyst [28]. This allows for a more durable catalyst that can perform efficiently over extended periods, particularly in the context of renewable energy applications such as unitized regenerative fuel cells (AEM-URFCs).

For example, Pt, Ni, and Co supported on Magnéli-phase titania have shown enhanced activity and stability for urea oxidation and ORR with their unique structural and electronic properties [29,30,31].

The functional properties of electrocatalysts are largely influenced by the synthesis method and the conditions under which they are prepared [32,33]. One of the most commonly used techniques is the sol–gel method, which allows for precise control over important factors such as particle size, composition, and surface area. This method involves converting metal salts or precursors into a gel, which is then processed to form nanoparticles [34,35,36].

Electrocatalysts produced using the sol–gel method exhibits excellent performance in reactions like oxygen reduction (ORR), urea oxidation, and hydrogenation. One of the key advantages of this technique is its ability to enhance the interaction between the metal particles and the support material, which is crucial for ensuring long-term stability and efficiency in electrochemical reactions [37,38,39].

The sol–gel method has proven to be especially effective for producing electrocatalysts based on platinum (Pt), nickel (Ni), and cobalt (Co). Numerous studies have explored the synthesis and performance of Pt, Ni, and Co-based electrocatalysts using the sol–gel method, with these materials supported on various substrates, including carbon black and Magnéli-phase titania [40,41,42]. For example, Pt supported on carbon black has shown superior activity in ORRs and better stability than conventional Pt catalysts, thanks to the high surface area and excellent electronic conductivity of carbon black [37,42]. Likewise, Ni and Co-based electrocatalysts on carbon black have demonstrated promising electrochemical performance in hydrogenation and urea electrooxidation reactions, particularly when supported on either carbon black or titania-based materials [43].

The flexibility of the sol–gel method in synthesizing hybrid Pt-Ni and Pt-Co electrocatalysts opens up further possibilities beyond just oxygen electrocatalysis. One particularly exciting opportunity is in CO_2_ electrochemical reduction (CO_2_RR), a key process for carbon-neutral energy solutions that converts CO_2_ into valuable fuels and chemicals. The sol–gel method allows for precise control over the composition, morphology, and dispersion of active sites, which could be beneficial for optimizing electrocatalysts for CO_2_RR.

Recent studies have shown that Pt-based materials combined with Ni or Co can effectively facilitate CO_2_ reduction by enhancing CO_2_ adsorption and proton transfer, while minimizing competing hydrogen evolution reactions (HERs) [44,45].

Additionally, the sol–gel method can be optimized by controlling factors such as precursor concentration, gelation conditions, and thermal treatment, allowing for the fine-tuning of the morphology, size, and electronic properties of metal nanoparticles [43]. This precise control results in highly active and durable electrocatalysts, which can withstand prolonged operation in fuel cells and other energy conversion devices.

In the current study, Pt-Co and Pt-Ni electrocatalysts were synthesized using the sol–gel method, supported on two distinct substrates: carbon black (Vulcan XC72R) and Magnéli-phase titania (N82). By dispersing Pt with Co and Ni on these supports, the research aims to assess the electrocatalytic performance of the resulting materials in oxygen evolution (OERs) and oxygen reduction reactions (ORRs). Through electrochemical analysis, the bifunctional capabilities of the electrocatalysts under alkaline conditions were evaluated, with a focus on their potential applications in unitized regenerative fuel cells (AEM URFCs). The combination of advanced synthesis techniques and tailored material compositions offers a promising route to improving the efficiency and durability of next generation electrocatalysts for renewable energy applications.

## 2. Results and Discussion

The structure and phase composition of the synthesized materials were examined by X-ray diffraction (XRD).

**Figure 1:**
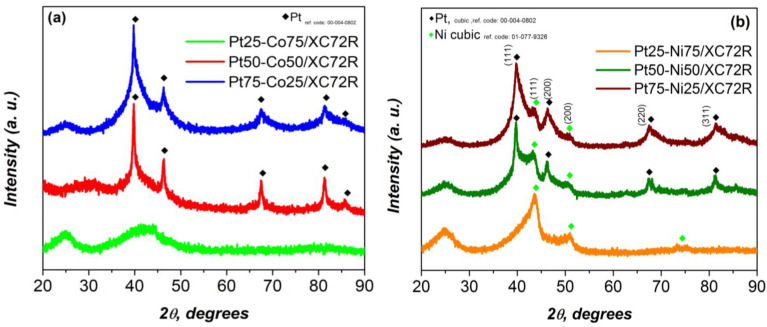
XRD data of prepared (**a**) PtX-CoX and (**b**) PtX-NiX electrocatalysts supported on XC72R.

Figure 1a presents the diffractograms of Pt/Co electrocatalysts with varying compositions in wt.%, deposited on an XC72R carbon support. The primary diffraction peak of platinum in the 75Pt/25Co and 50Pt/50Co samples is observed at a diffraction angle of 2θ ≈ 39.7°, followed by less pronounced peaks at 46.3°, 67.5°, 81.2°, and 85.8° 2θ, corresponding to the (111), (200), (220), and (311) crystallographic planes of platinum with a cubic structure (ref. code: 00-004-0802). The 25Pt/75Co sample exhibits an amorphous structure with no distinct diffraction peaks of Pt detected.

No distinct diffraction peaks corresponding to Co were observed in any of the samples analyzed. According to the crystallographic database, the main diffraction peak of the cubic Co phase is expected at 2θ ≈ 44.37° [ref. code: 00-001-1259], but it is not present in the diffractograms. Another primary peak of cubic Co could appear at 2θ ≈ 45.92° [ref. code: 00-088-2325], overlapping with the platinum peak. However, no peak shift was observed as the cobalt content increases to 50% (Pt50-Co50), ruling out the possibility of Co incorporation into the platinum structure. The absence of Co peaks can be explained by two factors: (1) the formation of amorphous Co due to the rapid evaporation of isopropanol during the sol–gel synthesis, and (2) the extremely fine dispersion of cobalt (crystallites smaller than 3–5 nm), which suppresses its diffraction signals. These factors are also responsible for the amorphous nature of the Pt25-Co75 material. Figure 1b presents the diffractograms of nanostructured Pt/Ni electrocatalysts deposited on an XC72R carbon support in various weight percentage ratios. The three diffractograms exhibit diffraction peaks that are characteristic of nickel at a diffraction angle of 2θ ≈ 44°, with the most intense peak observed in the sample containing 75% Ni. Additionally, weak diffraction signals are detected at approximately 2θ ≈ 51°. These peaks correspond to the (111) and (200) crystallographic planes of nickel with a cubic crystal structure.

A similar trend is observed for platinum, where the increase in Pt content results in the enhancement of the intensity of its characteristic diffraction peaks. In the samples with 75% and 50% Pt (75Pt/25Ni and 50Pt/50Ni), diffraction signals are clearly identified at 2θ ≈ 39.8°, 46.5°, 67.6°, and 81.3°, corresponding to the (111), (200), (220), and (311) planes of platinum with a cubic crystal structure. In contrast, no platinum peaks are registered in the sample containing 25% Pt and 75% Ni (25Pt/75Ni), as they are masked by the significantly more intense diffraction signals of nickel. The structural characterization is confirmed using reference cards for Pt (cubic, ref. code: 00-004-0802) and Ni (cubic, ref. code: 01-077-9326).

**Figure 2:**
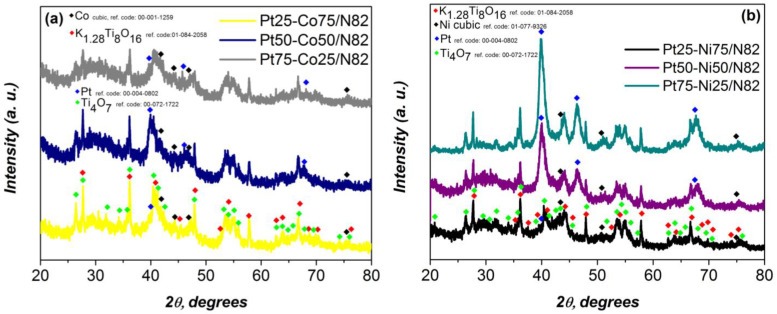
XRD data of prepared (**a**) PtX-CoX and (**b**) PtX-NiX electrocatalysts supported on N82.

Figure 2a presents the X-ray diffractograms of Pt/Co electrocatalysts with varying compositions, deposited on a non-carbon catalytic support based on Magnéli-phase titanium oxide (N82). The diffractograms reveal characteristic peaks for both platinum (Pt), with a primary diffraction maximum at 2θ = 39.9° (reference code: 00-004-0802), and cobalt (Co), with a corresponding primary peak at 2θ = 44.5° (reference code: 00-001-1259). The intensity of the diffraction peaks of Pt and Co is directly related to their respective concentrations in the catalysts.

When N82 is used as a catalytic support, the diffraction signals from the deposited metals appear to be less pronounced, as a significant portion of their peaks overlap with the intense reflections of the support material. The Magnéli-phase titanium oxide used as the support consists of two well-defined crystalline phases: Ti_4_O_7_ (reference code: 00-072-1722) and K_1 28_Ti_8_O_16_ (reference code: 01-084-2058).

Figure 2b presents the X-ray diffractograms of Pt-Ni electrocatalysts with varying compositions, deposited on a non-carbon catalytic support based onN82. The intensity of the diffraction peaks of Pt and Ni is directly correlated with the percentage of these metals in the electrocatalysts, consistent with the observations made in Figure 2a.

The efficiency of the sol–gel-prepared catalysts toward OER was evaluated in an aqueous solution of 1M of KOH at room temperature. The influence of the metal content (M1:M2) and the type of the electrocatalyst support on the electrocatalytic efficiency were studied with linear sweep voltammetry.

Previous studies have shown that Ni/MPT is a more effective electrocatalyst for the hydrogen evolution reaction (HER), while Co/MPT performs significantly better in the oxygen evolution reaction (OER), demonstrating exceptional stability under high anodic potentials [19,42].

**Figure 3:**
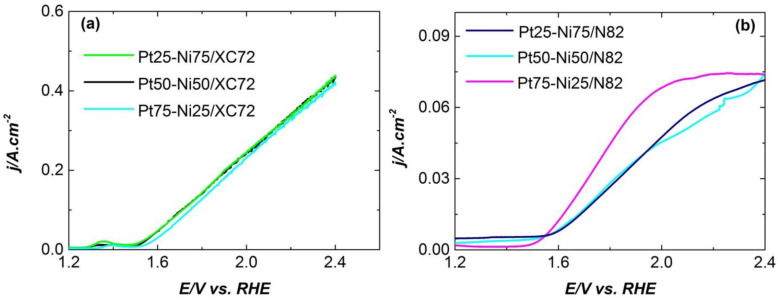
Polarization curves of OER on Pt-Ni- electrocatalysts with varying metal contents and types of support: (**a**) XC72 and (**b**) N82 recorded in 1M of KOH at room temperature; potential scan rate: 1 mV s^−1^.

Figure 3 illustrates the anodic polarization curves recorded for electrodes with PtX-NiX/N82 and PtX-NiX/XC72R, respectively.

The OER activity of PtX-NiX/N82 electrocatalysts is much lower compared to that of PtX-NiX/XC72R. The metal composition of the carbon-supported catalysts does not affect their OER efficiency, while when N82 is used, the intensity of the OER is lowest on the sample with the highest Pt content. The overpotential of ῃ _10mA·cm_^−1^ = 0.309 V was recorded for the PtX-NiX/XC72R samples and ῃ _10mA·cm_^−1^ = 0.363 V was recorded for the best performing MPT-based electrocatalyst, Pt75-Ni25/N82.

**Figure 4:**
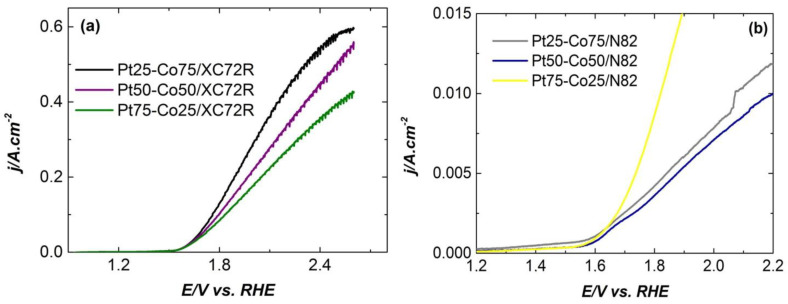
Polarization curves of PtX-CoX electrocatalysts supported on (**a**) XC72 and (**b**) N82 for OER recorded in 1M of KOH at room temperature; potential scan rate: 1 mV s^−1^.

The efficiency of the Pt-Co-based electrocatalysts toward OER was also examined. The results from Figure 4a show a decreasing of the OER intensity with increasing Pt content in the PtX-CoX/XC72R electrocatalysts following the order of Pt75-Co75/XC72R < Pt50-Co50/XC72R < Pt25-Co75/XC72R. This is not the case for the PtX-CoX/N82 electrocatalysts (Figure 4b) where the activity could not be related to the M1:M2 composition. Here, the obtained current densities were again much lower compared to PtX-CoX/XC72R electrocatalysts.

The overpotential of ῃ _10mA·cm_^−2^ = 0.350 V was recorded for the Pt25-Co75/XC72R samples and ῃ _10mA·cm_^−2^ = 0.591 V for the Pt75-Co25/N82.

The electrocatalysts’ activity toward OER in comparison with some state of the art electrocatalysts was presented in Table 1.

From the table, it is evident that the Pt25-Co75/XC72R sample demonstrates moderate OER activity compared to other catalysts. In contrast, the Pt75-Co25/N82 sample exhibits a higher overpotential of 0.591 V, indicating lower OER activity.

**Figure 5:**
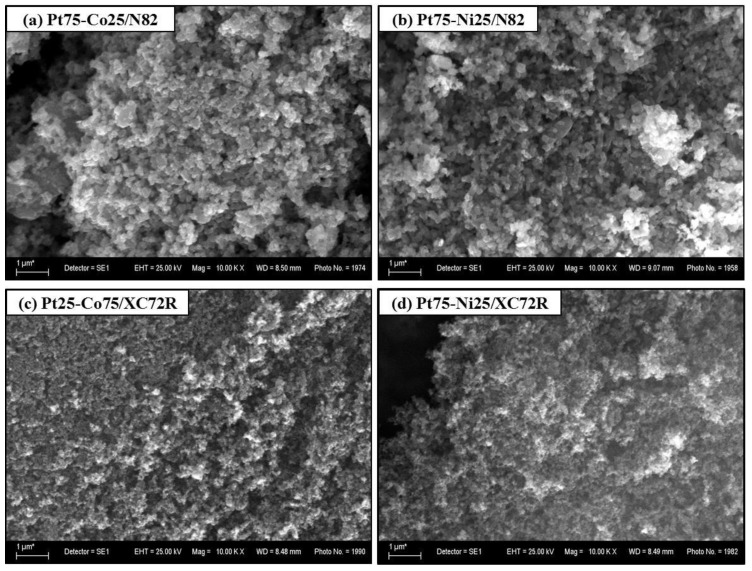
SEM images of the best performing electrocatalysts synthesized via the sol–gel method: (**a**) Pt75-Co25/N82, (**b**) Pt75-Ni25/N82, (**c**) Pt25-Co75/XC72R, and (**d**) Pt75-Ni25/XC72R.

SEM images of the best performing electrocatalysts are displayed in Figure 5a–d.

Distinct morphological differences between the two substrates are evident. The N82 matrix (Figure 5a,b) exhibits rounded rod-like structures that are characteristic for the Magnéli-phase titanium oxide particles with a size in the range of 100–150 nm. In contrast, the XC72R carbon matrix (Figure 5c,d) displays an amorphous structure composed of spherical particles and isolated agglomerates.

In both types of electrocatalysts, the metal particles are homogeneously dispersed across the substrate, although in some areas, the formation of larger agglomerates is observed.

Based on the performed preliminary electrochemical screening of the synthesized catalysts, Pt75-Co25/N82 and Pt25-Co75 samples were chosen for further RRDE testing of their ORR activity and proven bifunctionality in 0.5 M of KOH.

**Figure 6:**
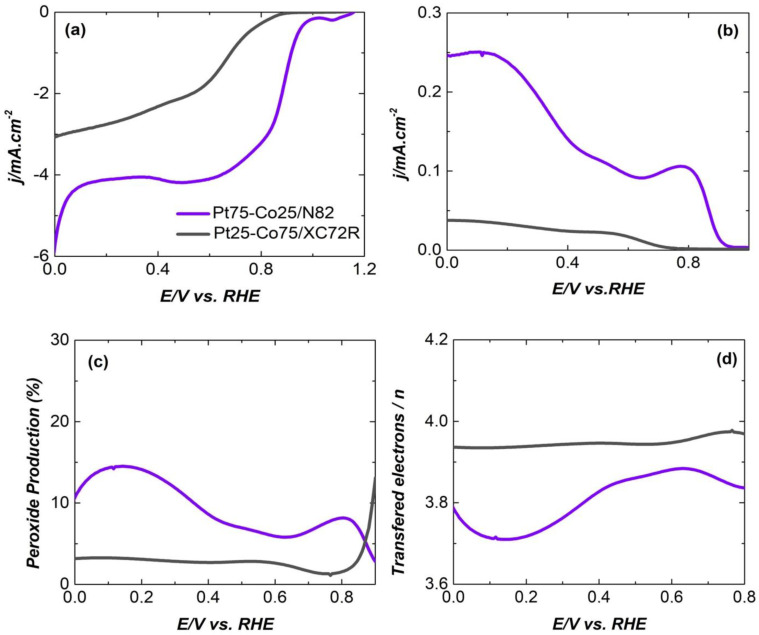
ORR activity of Pt75-Co25/N82 and Pt25-Co75/XC72R electrocatalysts; (**a**) LSVs at 5 mVs^−1^, (**b**) ring currents, (**c**) peroxide, and (**d**) electron transferred during ORR in 0.1 M of KOH with catalyst loading of 0.6 mg cm^−2^.

Using the rotating ring disk electrode (RRDE) methodology, the ORR activities of Pt75-Co25/N82 and Pt25-Co75/XC72R electrocatalysts were analyzed in an oxygen-saturated 0.5 M KOH electrolyte with the catalytic loading of 0.6 mg cm^−2^ and the observed trends are demonstrated in Figure 6. The recorded linear sweep voltammograms (LSVs) in Figure 6a exhibit the active nature of both electrocatalysts; however, the electrocatalytic performance of sample Pt25-Co75/XC72R is categorically superior. The onset potential (E_onset_), typically estimated at −0.1 mA·cm^−2^, came out to be −0.98 V vs. RHE which is remarkably higher compared to that of the Pt75-Co25/N82 sample (ca. 0.85 V vs. RHE). By applying a first derivative on the LSV, the halfwave potential (E_1/2_) of the sample Pt25-Co75/XC72R was estimated to be 0.98 V vs. RHE, further endorsing the excellent kinetics of ORR launched by Pt25-Co75/XC72R.

On the other hand, the E1/2 of the sample Pt75-Co25/N82 was 0.67 V vs. RHE. The ring current density of sample Pt25-Co75/XC72R presented an incessant rise soon after crossing the ORR E_onset_ whereas sample Pt75-Co25/N82 remained relatively stable. The offshoot of this observation can be noticed in the peroxide production (Figure 6c) where sample Pt25-Co75/XC72R exhibited a slightly higher peroxide yield with an increasing trend (ca. 5–15%) while the peroxide yield for sample Pt75-Co25/N82 remained well below 5%. However, the electron transfer number for both electrocatalysts remained above 3.5 throughout the potential window, illustrating a direct 4-electron ORR.

**Figure 7:**
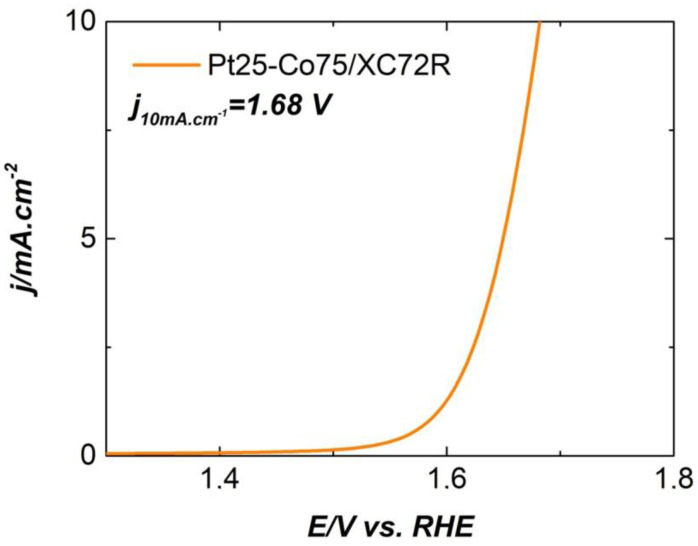
LSV curve of Pt25-Co75/XC72R electrocatalyst on RRDE in 0.1 M of KOH with catalyst loading of 0.6 mg cm^−2^.

The Bifunctional Index (the variance in OER and ORR metrics) of the Pt25-Co75/XC72R was as calculated according Equation (1):

ΔE = E_j=10_ − E_1/2_ (1)

here E_1/2_ is the halfwave potential (E_1/2_) from the LSV curve in Figure 6a and E_j=10_ is the OER potential taken at a current density of 10 mA·cm^−2^ as taken from Figure 7.

The ΔE value for Pt25-Co75/XC72R is 0.7 V, which shows its superior bifunctional activity (OER/ORR). A similar value has been reported for ironpolyphthalocyanine metallic–organic frameworks (MOFs) over the carbon black matrix (FePPc@CB) (0.68 V) [55] and hybrid Co/CoO/Ce-Doped WO_3_ nanoparticles on a ZIF-L Framework (0.679 V) [56] with the application in Zink—Air Batteries.

The validation of the bifunctionality of the examined electrocatalysts in real AEM-URFC is under study.

## 3. Conclusions

In this study, hybrid Pt-Ni and Pt-Co electrocatalysts were successfully synthesized using the sol–gel method over substrates with different chemical natures (carbon and Magnelli-phase titania). The electrochemical performance of these electrocatalysts was systematically evaluated, revealing key insights into their activity and potential applications.

The carbon-based Pt-Ni electrocatalysts demonstrated superior activity for oxygen evolution reaction (OER) compared to their MPT-based counterparts. Similarly, the carbon-based Pt-Co electrocatalysts exhibited enhanced activity toward OER and ORR, as confirmed by RRDE measurements.

Among the studied materials, the electrocatalysts Pt25-Co75/XC72 and Pt75-Co25/N82 showed promising bifunctional activity for oxygen electrocatalysis (OER/ORR). Based on the promising bifunctional activity observed in Pt25-Co75/XC72 and Pt75-Co25/N82 in our study, these materials could be explored for CO_2_RR applications.

Further studies exploring the structural and electronic modifications of these materials for CO_2_RR could expand their applicability in sustainable energy conversion and carbon-neutral technologies.

Overall, the synthesized electrocatalysts demonstrate potential for application in both water electrolysis and fuel cell modes within an anion exchange membrane-unitized regenerative fuel cell (AEM-URFC) system, highlighting their versatility and practical significance in sustainable energy conversion technologies.

## 4. Materials and Methods

### 4.1. Sol–Gel Synthesis of Electrocatalysts

In this work, bimetal composite electrocatalysts were prepared via the sol–gel process using acetylacetonate precursors (M((C_5_H_7_O_2_)_n_)_m_, (M = Ni, Co, Pt) (Alpha Acer)) on two supports—Magneli-phase titanium oxide (with the general formula of Ti_n_O_2n−1_, commercially available from Ti-dynamics Co., Ltd., Changsha, China, designated N82) and upon carbon black (Vulcan XC-72, designated as XC72).

**Figure 8:**
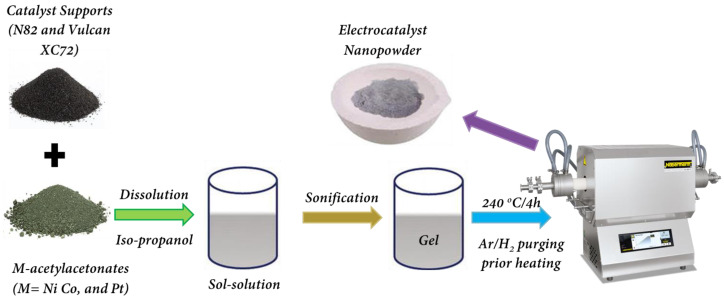
Schematic representation of synthesis of electrocatalysts.

**Figure 9:**
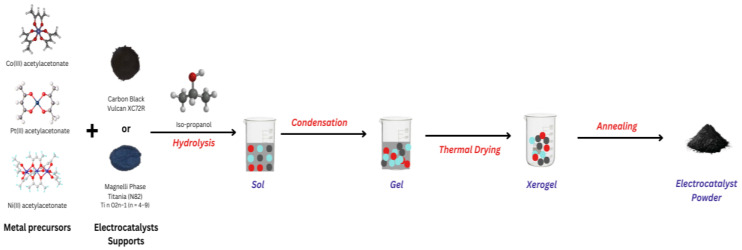
Proposed mechanism of sol–gel synthesis of M_1_X-M_2_X/N82 and M_1_X-M_2_X/XC72 electrocatalysts.

The content of the metal part (Co, Ni, and Pt) varied within the range of 25–75 wt.%. The test samples were denoted as M_1_X-M_2_X/N82 and M_1_X-M_2_X/XC72, respectively, where M_1_ = Co or Ni,M_2_ = Pt, and X is the metal content in wt.%. Initially, the metal acetylacetonate powders and the supports were dissolved in a defined amount of isopropanol at room temperature and mixed using a magnetic stirrer. To achieve better homogenization, the samples were treated in an ultrasonic bath (60 °C) for approximately 30 min until gelation occurred. The gels were heated in a tube furnace in a reducing atmosphere to 240 °C for 4 h (a temperature rate of 2 °C min^−1^ (2 h), and then the temperature was maintained for 2 h). The samples were taken out after complete cooling to room temperature. The obtained electrocatalysts were homogenized in agate mortar and stored in a dry oxygen-free atmosphere (Figure 8 and Figure 9).

### 4.2. Electrodes Fabrication

**Figure 10:**
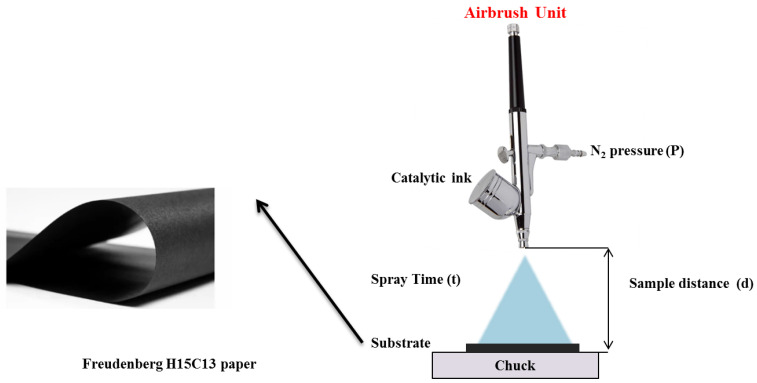
Schematic representation of the electrodes’ preparation.

The catalytic inks were prepared with calculated amounts of catalyst powders (M1X-M2X/N82 and M1X-M2X/XC72), DI water (18.2 MΩ, Milli-Q), 2-propanol (NPA, Sigma-Aldrich, St. Louis, MI, USA), and Nafion™ (5 wt.% solution, 1100 EW, Ion Power, New Castle, DE, USA). The catalyst inks were mixed for 5 min by sonication in a bath sonicator. The as-prepared inks were loaded in the airbrush unit for spray deposition on the surface of a Freudenberg FCCTKG H2315 gas-diffusion layer (Figure 10). The electrocatalysts were deposited as a thin layer with a fixed loading of 0.5 mg cm^−2^.

### 4.3. Electrocatalyst Characterization

#### 4.3.1. Physicochemical Characterization

The structural features of the as-prepared electrocatalysts were characterized by X-ray diffraction (XRD) via a Philips ADP15 X-ray diffractometer with Cu-Kα radiation (λ = 1. 54,178 Å) at a constant rate of 0. 02° s^−1^ over an angle range of 4° to 80° 2θ. For the interpretation of the results, the database PDF 2—2022, ICD was used.

The morphology, structure, and composition of the electrocatalysts were also investigated by scanning electron microscopy (SEM)/Energy Dispersive Spectroscopy (EDS) using a JEOL JSM 6390 electron microscope (JEOL, Tokyo, Japan) equipped with an INCA Oxford elemental detector (Oxford Instruments, London, UK).

#### 4.3.2. Electrochemical Characterization

i Initial OER activity testing

The initial electrochemical screening of the electrocatalysts/electrodes was conducted at room temperature in argon saturated with 25% KOH (pH = 14) in a standard three-electrode electrochemical cell (equipped with a Luggin capillary, whose tip was set at a distance of 1–2 mm from the surface of the working electrode for the minimization of the iR drop) utilizing a Pt wire as the counter electrode and a saturated Ag/AgCl electrode as the reference electrode. All potentials were converted and reported with respect to the reversible hydrogen electrode (RHE) (E_RHE_ = E_Measured_ + Eo_Ag/AgCl_ + 0.059 × pH at 25 °C).

For electrochemical evaluation of the sol–gel-synthesized electrocatalyst, the conventional electrochemical techniques of cycling voltammetry and quasi-steady-state polarization tests were applied.

In order to better understand the effect of the type of support and the metal composition of the processes that occurred on the electrode surface, the cyclic voltammograms (CVs) were recorded in the potential range between the hydrogen and oxygen evolution at scan rate of 100 mV s^−1^. The quasi-steady-state polarization tests (LSV) of OER and HER activities were carried out in a potentiodynamic mode with a scan rate of 1 mV s^−1^. All linear sweep voltammetry (LSV) curves were acquired without iR compensation.

ii ORR and OER testing with RRDE

The electrochemical investigations were carried out in a typical three-electrode configuration comprising the working electrode, the graphite rod as the counter electrode, and a Ag/AgCl electrode as a reference. The electrochemical data were collected through a Pine WaveVortex Rotating Disk Electrode (RDE) system connected with a Pine potentiostat. For the electrochemical analyses, first inks were prepared by dispersing 5 mg of electrocatalysts in a mixture of 985 µL isopropanol (Alfa Aesar, Haverhill, MA, USA) and 15 μL of Nafion^®^ D-520 (5 wt.%, Alfa Aesar) and then the obtained suspension was subjected to a probe sonicator for 20 min. Afterward, the ink-containing gas vials were transferred to an ultrasonic bath sonicator for further homogenization for the next 30 min under ambient conditions. For the ORR, the working electrode was fabricated by drop-casting the inks onto a rotating ring disk electrode (RRDE, E6R2 Series, PINE research, Durham, DC, USA) with 0.6 mg cm^−2^ of electrocatalyst loading whereas for the OER measurements, the working electrode was based on RDE (E5 Series PINE research, Durham, USA) with the same electrocatalyst loading. An in-house-prepared 0.1 M KOH solution was used as an electrolytic media. The ORR measurements were carried out in the alkaline electrolyte saturated with ultra-pure O_2_; however, during OER, the electrolyte was purged with pure N2. To report, the potential values were converted with respect to the reversible hydrogen potential (RHE) using Equation (2):

E_(RHE)_ = E_(Ag/AgCl)_ + 0.0591 × pH + E°_(Ag/AgCl)_ (2)

The potential window for the ORR was kept between 1.23 and 0 V vs. RHE while keeping the ring current constant at 1.23 V vs. RHE. Before recording the actual linear sweep voltammograms (LSVs) at 5 mVs^−1^, the electrocatalyst was conditioned by applying multiple cyclic voltammetry (CV) until a reproducible trend was observed at the scan rate of 50 mVs^−1^ (this step remains the same for both ORR and OER). Eventually, the peroxide yield (%) and the number of electrons transferred (n) during ORR were estimated by recording the disk current (I_disk_) and the ring current (I_ring_) as given in Equations (3) and (4), respectively. In Equations (2) and (3), ‘N’ is the collection efficiency (38%) of the RRDE with the Pt ring having a surface area of 0.2356 cm^2^.

Peroxide yield (%) = (200 × I_ring_/N)/(I_disk_ + I_ring_/N) (3)

n = (4 I_disk_)/(I_(disk)_ + I_ring_/N) (4)

The potential window for OER was maintained at 1.23 and 2.23 V vs. RHE. It should be noted that the ORR and OER were performed under hydrodynamic conditions with 1600 rpm rotation rates of RRDE/RDE.

## Figures and Tables

**Table 1:** Selected electrocatalysts for the OER in an alkaline solution.

Electrocatalysts Composition	Overpotential (V)	Reference
Pt_25_-Co_75_/XC72R(deposited on Freudenberg FCCTKG H2315)	0.350	In this work
Pt_75_-Co_25_/N82(deposited on Freudenberg FCCTKG H2315	0.591	In this work
NiCo_2_O_4_ (deposited on Ni foam)	0.250	[46]
NiFeCrO_4_ (deposited on Ni plate)	0.285	[47]
CoFe_2_O_4_ (deposited on glassy carbon electrode)	0.370	[48]
Co_3_O_4_ (deposited on Au electrode)	0.400	[49]
CoCr_2_O_4_/CNT (deposited on glassy carbon electrode)	0.326	[50]
Cu(dto)/C	0.400	[51]
NiCoO (deposited on FTO)	0.460	[52]
N-doped multi-walled carbon nanotubes (NMWNT)	0.320	[53]
N and S-doped graphene on graphite foam (SNG@GF)	0.330	[53]
S-NiFeOOH@Fe-Ni_3_S_2_ on NiFe foam	0.242	[54]

## Data Availability

The article contains the original contributions made in this study. As part of the current investigation, the corresponding author can provide the data described in this publication upon request because they are part of the ongoing study.

## Abbreviations

The following abbreviations are used in this manuscript:

MPT	Magnelli-phase titania
HER	Hydrogen evolution reaction
OER	Three letter acronym
ORR	Oxygen reduction reaction
HOR	Hydrogen oxidation reaction
LSV	Linear sweep voltammetry
CV	Cycling voltammetry
RRDE	Rotating disk electrode
CO_2_RR	CO_2_ electrochemical reduction

## References

[1] Bard A.J., Fox M.A. (1995). Artificial photosynthesis: Solar splitting of water to hydrogen and oxygen. Acc. Chem. Res..

[2] Lewis N.S., Nocera D.G. (2006). Powering the planet: Chemical challenges in solar energy utilization. Proc. Natl. Acad. Sci. USA.

[3] Nocera D.G. (2009). Chemistry of personalized solar energy. Inorg. Chem..

[4] Zhao S., Yan L., Luo H., Mustain W., Xu H. (2018). Recent progress and perspectives of bifunctional oxygen reduction/evolution catalyst development for regenerative anion exchange membrane fuel cells. Nano Energy.

[5] Wang Y., Leung D.Y.C., Xuan J., Wang H. (2017). A review on unitized regenerative fuel cell technologies, part B: Unitized regenerative alkaline fuel cell, solid oxide fuel cell, and microfluidic fuel cell, Renew. Sustain. Energy Rev..

[6] Gabbasa M., Sopian K., Fudholi A., Asim N. (2014). A review of unitized regenerative fuel cell stack: Material, design and research achievements. Int. J. Hydrogen Energy.

[7] Sadhasivam T., Dhanabalan K., Roh S.H., Kim T.H., Park K.W., Jung S. (2017). A comprehensive review on unitized regenerative fuel cells: Crucial challenges and developments. Int. J. Hydrogen Energy.

[8] Kanan M.W., Nocera D.G. (2008). In situ formation of an oxygen-evolving catalyst in neutral water containing phosphate and Co^2+^. Science.

[9] Gorlin Y., Jaramillo T.F. (2010). A bifunctional nonprecious metal catalyst for oxygen reduction and water oxidation. J. Am. Chem. Soc..

[10] Chen G., Bare S.R., Mallouk T.E. (2002). Development of supported bifunctional electrocatalysts for unitized regenerative fuel cells. J. Electrochem. Soc..

[11] Nørskov J.K., Bligaard T., Logadottir A., Kitchin J.R., Chen J.G., Pandelov S., Stimming U. (2005). Trends in the exchange current for hydrogen evolution. J. Electrochem. Soc..

[12] Debe M.K. (2012). Electrocatalyst approaches and challenges for automotive fuel cells. Nature.

[13] Greeley J., Stephens I.E.L., Bondarenko A.S., Johansson T.P., Hansen H.A., Jaramillo T.F., Rossmeisl J., Chorkendorff I., Nørskov J.K. (2009). Alloys of platinum and early transition metals as oxygen reduction electrocatalysts. Nat. Chem..

[14] Strmcnik D., Uchimura M., Wang C., Subbaraman R., Danilovic N., van der Vliet D., Paulikas A.P., Stamenkovic V.R., Markovic N.M. (2013). Improving the hydrogen oxidation reaction rate by promotion of hydroxyl adsorption. Science.

[15] Rajendran S., Naushad M., Raju K., Boukherroub R. (2019). Emerging Nanostructured Materials for Energy and Environmental Science.

[16] Belenov S., Pavlets A., Paperzh K., Mauer D., Menshikov V., Alekseenko A., Pankov I., Tolstunov M., Guterman V. (2023). The PtM/C (M = Co, Ni, Cu, Ru) Electrocatalysts: Their Synthesis, Structure, Activity in the Oxygen Reduction and Methanol Oxidation Reactions, and Durability. Catalysts.

[17] Sui S., Wang X., Zhou X. (2017). A comprehensive review of Pt electrocatalysts for the oxygen reduction reaction: Nanostructure, activity, mechanism and carbon support in PEM fuel cells. J. Mater. Chem. A.

[18] Zhang J., Xia Z., Dai L. (2015). Carbon-based electrocatalysts for advanced energy conversion and storage. Sci. Adv..

[19] Maksimova K., Lefterova E., Slavcheva E. (2015). Nanostructured nickel and cobalt supported on Magneli—Phase titania—Preparation, properties and catalytic efficiency toward alkaline water electrolysis. Nanosci. Nanotechnol..

[20] Kim J.H., Lee S.Y., Lee H.J. (2025). Strategies for the Design and Synthesis of Pt-Based Nanostructured Electrocatalysts in Proton Exchange Membrane Fuel Cells (PEMFCs). ACS Eng. Au.

[21] Touni A., Grammenos O., Banti A., Karfaridis D., Prochaska C., Lambropoulou D., Pavlidou E., Sotiropoulos S. (2021). Iridium oxide-nickel-coated titanium anodes for the oxygen evolution reaction. Electrochim. Acta.

[22] Fan Y., Feng X., Zhou W., Murakami S., Kikuchi K., Nomura N., Wang L., Jiang W., Kawasaki A. (2018). Preparation of monophasic titanium sub-oxides of Magnéli phase with enhanced thermoelectric performance. J. Eur. Ceram. Soc..

[23] Liu H., Xiao H., Qiao Y., Luo M.Q., Wang C., Yang L.X., Zeng C.L., Fu C. (2023). Preparation, characterization, and electrochemical behavior of a novel porous Magnéli phase Ti_4_O_7_-coated Ti electrode. Ceram. Int..

[24] Kim M., Choi J., Lee W., Ahn Y.Y., Lee H., Cho K., Lee J. (2023). Performance of Magnéli phase Ti_4_O_7_ and Ti ^3+^ self-doped TiO_2_ as oxygen vacancy-rich titanium oxide anodes: Comparison in terms of treatment efficiency, anodic degradative pathways, and long-term stability. Appl. Catal. B Environ..

[25] Slavcheva E., Nikolova V., Petkova T., Lefterova E., Dragieva I., Vitanov T., Budevski E. (2005). Electrocatalytic activity of Pt and PtCo deposited on Ebonex by BH reduction. Electrochim. Acta.

[26] Figueiredo W., Prakash R., Vieira C.G., Lima D.S., Carvalho V.E., Soares E.A., Buchner S., Raschke H., Perez-Lopez O.W., Baptista D.L. (2022). New insights on the electronic factor of the SMSI effect in Pd/TiO_2_ nanoparticles. Appl. Surf. Sci..

[27] Bertella F., Concepción P., Martínez A. (2017). TiO_2_ polymorph dependent SMSI effect in Co-Ru/TiO_2_ catalysts and its relevance to Fischer-Tropsch synthesis. Catal. Today.

[28] Dogan D.C., Choi J., Seo M.H., Lee E., Jung N., Yim S.-D., Yang T.-H., Park G.-G. (2021). Enhancement of Catalytic Activity and Durability of Pt Nanoparticle through Strong Chemical Interaction with Electrically Conductive Support of Magnéli Phase Titanium Oxide. Nanomaterials.

[29] Ekanayake A., Mai H., Chen D., Caruso R.A. (2025). Recent advances in synthesis and application of Magnéli phase titanium oxides for energy storage and environmental remediation. Chem. Sci..

[30] Augustyn V., Simonbc P., Dunn B. (2014). Pseudocapacitive oxide materials for high-rate electrochemical energy storage. Energy Environ. Sci..

[31] Wu Q.M., Ruan J.M., Zhou Z.C., Sang S.B. (2015). Magneli phase titanium sub-oxide conductive ceramic Ti_n_O_2n−1_ as support for electrocatalyst toward oxygen reduction reaction with high activity and stability. J. Cent. South Univ..

[32] Gayen P., Saha S., Liu X., Sharma K., Ramani V.K. (2021). High-performance AEM unitized regenerative fuel cell using Pt-pyrochlore as bifunctional oxygen electrocatalyst. Proc. Natl. Acad. Sci. USA.

[33] Wang Y., Leung D.Y.C., Xuan J., Wang H. (2016). A review on unitized regenerative fuel cell technologies, part-A: Unitized regenerative proton exchange membrane fuel cells. Renew. Sust. Energy.

[34] Bokov D., Turki Jalil A., Chupradit S., Suksatan W., Javed Ansari M., Shewael I.H., Valiev G.H., Kianfar E. (2021). Nanomaterial by Sol-Gel Method: Synthesis and Application. Adv. Mater. Sci. Eng..

[35] Davis R.J., Mayer J.W. (2016). Sol-Gel Synthesis of Nanostructured Catalysts. Catal. Today.

[36] Omeiza L.A., Abdalla A.M., Wei B., Dhanasekaran A., Subramanian Y., Afroze S., Reza M.S., Bakar S.A., Azad A.K. (2023). Nanostructured Electrocatalysts for Advanced Applications in Fuel Cells. Energies.

[37] De A., Kim M.S., Adhikari A., Patel R., Kundu S. (2024). Sol–gel-derived nanostructured electrocatalysts for oxygen evolution reaction: A review. J. Mater. Chem. A.

[38] Letchumanan I., Yunus R.M., Masdar M.S., Karim N.A. (2025). Advancements in electrocatalyst architecture for enhanced oxygen reduction reaction in anion exchange membrane fuel cells: A comprehensive review. Int. J. Hydrogen Energy.

[39] Bari G.A.K.M.R., Jeong J.-H. (2024). Comprehensive Insights and Advancements in Gel Catalysts for Electrochemical Energy Conversion. Gels.

[40] Ren X., Lv Q., Liu L., Liu B., Wang Y., Liu A., Wu G. (2020). Current progress of Pt and Pt-based electrocatalysts used for fuel cells. Sustain. Energ. Fuels.

[41] Maksimova-Dimitrova K., Mladenova B., Borisov G., Slavcheva E. (2024). Ni and Co Catalysts on Interactive Oxide Support for Anion Exchange Membrane Electrolysis Cell (AEMEC). Inorganics.

[42] Zhao X., Li J., Zhang J., Yang J.H. (2023). Urea electrooxidation: Research progress and application of supported nickel-based catalysts. Ionics.

[43] Kamp E., Thielert H., von Morstein O., Kureti S., Schreiter N., Repke J.U. (2020). Investigation on the simultaneous removal of COS, CS_2_ and O_2_ from coke oven gas by hydrogenation on a Pd/Al_2_O_3_ catalyst. Catal. Sci. Technol..

[44] Albertini P.P., Newton M.A., Wang M., Lecina O.S., Green P.B., Stoian D.C., Oveisi E., Loiudice A., Buonsanti R. (2024). Hybrid oxide coatings generate stable Cu catalysts for CO_2_ electroreduction. Nat Mater..

[45] Koolen C.D., Luo W., Züttel A. (2023). From Single Crystal to Single Atom Catalysts: Structural Factors Influencing the Performance of Metal Catalysts for CO_2_. ACS Catal..

[46] Wang L., Gu C., Ge X., Zhang J., Zhu H., Tu J. (2017). Anchoring Ni_2_P Sheets on NiCo_2_O_4_ Nanocone Arrays as Optimized Bifunctional Electrocatalyst for Water Splitting. Adv. Mater. Interfaces.

[47] Singh R.N., Singh J.P., Lal B., Thomas M.J., Bera S. (2006). New NiFe_2_−xCr_x_O_4_ Spinel Films for O_2_ Evolution in Alkaline Solutions. Electrochim. Acta.

[48] Li M., Xiong Y., Liu X., Bo X., Zhang Y., Han C., Guo L. (2015). Facile Synthesis of Electrospun MFe_2_O_4_ (M = Co, Ni, Cu, Mn) Spinel Nanofibers with Excellent Electrocatalytic Properties for Oxygen Evolution and Hydrogen Peroxide Reduction. Nanoscale.

[49] Koza J.A., He Z., Miller A.S., Switzer J.A. (2012). Electrodeposition of Crystalline Co_3_O_4_—A Catalyst for the Oxygen Evolution Reaction. Chem. Mater..

[50] Al-Mamun M., Su X., Zhang H., Yin H., Liu P., Yang H., Wang D., Tang Z., Wang Y., Zhao H. (2016). Strongly Coupled CoCr_2_O_4_/Carbon Nanosheets as High Performance Electrocatalysts for Oxygen Evolution Reaction. Small.

[51] Putra R.P., Horino H., Rzeznicka I.I. (2020). An Efficient Electrocatalyst for Oxygen Evolution Reaction in Alkaline Solutions Derived from a Copper Chelate Polymer via In Situ Electrochemical Transformation. Catalysts.

[52] Dure A.A., Nazir N.A., Haider A., Iqbal M., Alwadai N., Kausar A., Ahmad A. (2023). Fabrication of Efficient Electrocatalysts for Electrochemical Water Oxidation Using Bimetallic Oxides. ACS Omega.

[53] Stelmachowski P., Duch J., Sebastián D., Lázaro M.J., Kotarba A. (2021). Carbon-Based Composites as Electrocatalysts for Oxygen Evolution Reaction in Alkaline Media. Materials.

[54] Niu S., Tang T., Qu Y., Chen Y., Luo H., Pan H., Jiang W.-J., Zhang J., Hu J.-S. (2024). Mitigating the Reconstruction of Metal Sulfides for Ultrastable Oxygen Evolution at High Current Density. CCS Chem..

[55] Cheng W.Z., Liang J.L., Yin H.-B., Wang Y.-J., Yan W.-F., Zhang J.-N. (2020). Bifunctional iron-phtalocyanine metal–organic framework catalyst for ORR, OER and rechargeable zinc–air battery. Rare Met..

[56] Zhang Y., Ma X., Zhu K., Wang J., Cheng Z., Li G., Yang L., Bai Z. (2023). Hybrid Co/CoO/Ce-Doped WO_3_ Nanoparticles on a ZIF-L Framework as Bifunctional Oxygen Electrocatalysts for Rechargeable Zinc–Air Batteries. ACS Publ. J. Contrib..

